# Microbial electrolysis: a promising approach for treatment and resource recovery from industrial wastewater

**DOI:** 10.1080/21655979.2022.2051842

**Published:** 2022-03-17

**Authors:** Yamini Koul, Viralkunvar Devda, Sunita Varjani, Wenshan Guo, Huu Hao Ngo, Mohammad J. Taherzadeh, Jo-Shu Chang, Jonathan W. C. Wong, Muhammad Bilal, Sang-Hyoun Kim, Xuan-Thanh Bui, Roberto Parra-Saldívar

**Affiliations:** a Paryavaran Bhavan, Gujarat Pollution Control Board, Gandhinagar, India; b School of Environment and Sustainable Development, Central University of Gujarat, Gandhinagar, India; c Centre for Technology in Water and Wastewater, School of Civil and Environmental Engineering, University of Technology Sydney, Sydney, Australia; d Swedish Centre for Resource Recovery, University of Borås, Borås, Sweden; e Department of Chemical Engineering, National Cheng Kung University, Tainan, Taiwan; f Institute of Bioresource and Agriculture and Department of Biology, Hong Kong Baptist University, Hksar, Hong Kong; g School of Life Science and Food Engineering, Huaiyin Institute of Technology, Huaian, China; h School of Civil and Environmental Engineering, Yonsei University, Seoul, Republic of Korea; i Faculty of Environment and Natural Resources, Ho Chi Minh City University of Technology (Hcmut), Ho Chi Minh City, Vietnam; j Key Laboratory of Advanced Waste Treatment Technology, Vietnam National University Ho Chi Minh (Vnu-hcm), Ho Chi Minh City, Vietnam; k Escuela de Ingeniería y Ciencias- Centro de Biotecnología-FEMSA, Tecnológico de Monterrey, Campus Monterrey, Mexico

**Keywords:** Industrial effluents, electrochemical technology, anaerobic digestion, resources, environmental sustainability

## Abstract

Wastewater is one of the most common by-products of almost every industrial process. Treatment of wastewater alone, before disposal, necessitates an excess of energy. Environmental concerns over the use of fossil fuels as a source of energy have prompted a surge in demand for alternative energy sources and the development of sophisticated procedures to extract energy from unconventional sources. Treatment of municipal and industrial wastewater alone accounts for about 3% of global electricity use while the amount of energy embedded in the waste is at least 2–4 times greater than the energy required to treat the same effluent. The microbial electrolysis cell (MEC) is one of the most efficient technologies for waste-to-product conversion that uses electrochemically active bacteria to convert organic matter into hydrogen or a variety of by-products without polluting the environment. This paper highlights existing obstacles and future potential in the integration of Microbial Electrolysis Cell with other processes like anaerobic digestion coupled system, anaerobic membrane bioreactor and thermoelectric micro converter.

## Graphical abstract_R1

**Figure:**
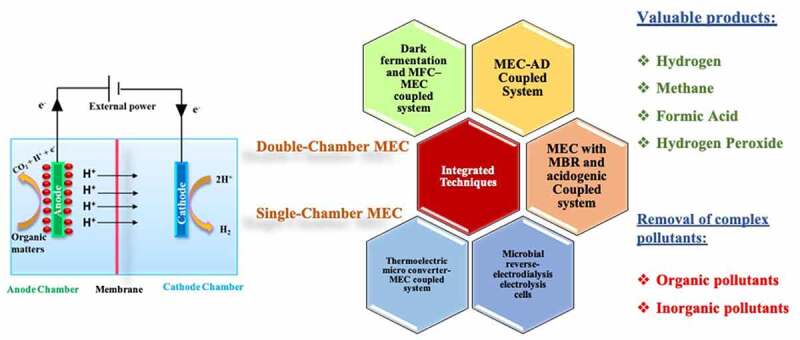

## 1. Introduction

With an ever-increasing population of the world, meeting the energy requirement of such a vast population has posed to be the single most critical challenge to mankind [1,2]. The requirement for energy has continued to go up exponentially as the population has grown over the years [3]. However, our reliance on fossil fuels to generate the majority of our energy has not altered, and we continue to utilize fossil fuels such as coal, oil, and others to meet our needs [4] which eventually results in the release of pollutants into the environment, necessitating further remediation [5,6, 63]. A lot of energy is used to run a number of industries around the globe, leaving behind by-products like wastewater [7]. The different wastes generated in such processes require treatment before disposal which in turn requires a lot of energy. Thus, the production and treatment energy nexus continues [8]. Wastewater is one of the most abundant by-products of practically all industries. Treatment of wastewater alone, before its disposal, requires a surplus amount of energy [9]. Apart from industries, a lot of municipal wastewater is generated on a daily basis which also requires energy for its treatment. About 3% of the global electricity consumption is accounted for the treatment of municipal wastewater [10]. The environmental concerns arising from the use of fossil fuels as the source of energy have also led to the increased demand for alternative sources of energy and the refined processes to harness the energy from such unconventional sources [11–13]. The energy embedded in the wastewater is at least 2–4 times more than the amount of energy required for the treatment of the same wastewater. Therefore, attempts must be directed toward developing and refining such technologies that can produce energy from sources otherwise deemed waste [14]. Additionally, other value-added products like chemicals, metals, clean water, etc. can be extracted from wastewater stream to maximize productivity and efficiency of the facility [15]. The composition of wastewater greatly depends upon the source of generation [16]. Wastewater is generally a mixture of various organic and inorganic components which can generate by-products like H_2_ during treatment. The different sources of wastewater can be domestic wastewater, landfill wastewater, industrial wastewater, refinery wastewater, livestock, and dairy wastewater to name a few [17]. The microbes and treatment technology used greatly depend on the substrate present in the wastewater. Similarly, end product derived from treatment of wastewater also depends upon the substrate. Ditzig et al. demonstrated the production of hydrogen using the domestic wastewater as a substrate using the MECs along with the treatment of wastewater which resulted in the reduction of BOD and COD [18].

Among the most efficient technology for the process of waste to product conversion is the Microbial Electrolysis Cell [19,20]. The MEC setup uses the exoelectronic microbes to convert the biodegradable matter like the organic matter present in the waste stream into electric current and protons [21,22]. The microbes are present at the anode and act upon the biodegradable waste to generate electricity and protons. The electrons are then transferred to the cathode where they reduce the protons for H_2_ production [23,24]. The type of microorganism used in the MEC depends on the substrate. *For Aeromonas hydrophila, Thermincola sp, Geothrix fermentans* and *Gluconobacter oxydans* substrate is acetate; for *S. putrefaciens* and *S, oneidensis* substrate is acetate lactate while for *K. pneumoniae, Rhodoferax ferrireducens* and *E cloacae* substrate is acetate glucose [25].

The microbial electrolysis is an endothermic process and therefore for the production of H_2_ at the cathode, a small voltage is applied between the two electrodes to forcefully initiate the current generation. However, the applied voltage is only about 0.2–0.8 V and can be supplied by low-grade microbial fuel cells or small solar panels [26]. The production of H_2_ using MECs has not only been hailed for commercialization because of better H_2_ production performance in comparison to the other H_2_ technologies like fermentation but also because of its higher performance efficiency than other microbial electrochemical systems like MFCs etc [26].

This review aims to explore MEC applications in the following instances: an overview of Microbial Electrolysis cells for energy generation and recycling, such as hydrogen, methane, and formic acid; contaminant removal, particularly complex organic and inorganic pollutants; and resource recovery. In this review new MEC technology concepts are discussed, such as coupling with other technologies for value-added applications such as MEC-anaerobic digestion, MEC with anaerobic Membrane Bioreactor (MBR) and acidogenic, MEC-Thermoelectric micro converter, Dark fermentation, and Microbial Fuel Cell (MFC)–MEC, and MEC with Microbial reverse-electrodialysis electrolysis cell systems. Finally, challenges, prospects, and the life cycle assessment of the process are discussed.

## 2. Types of the microbial electrolysis system

### 2.1 Double-chamber MECs system

**Figure 1.**
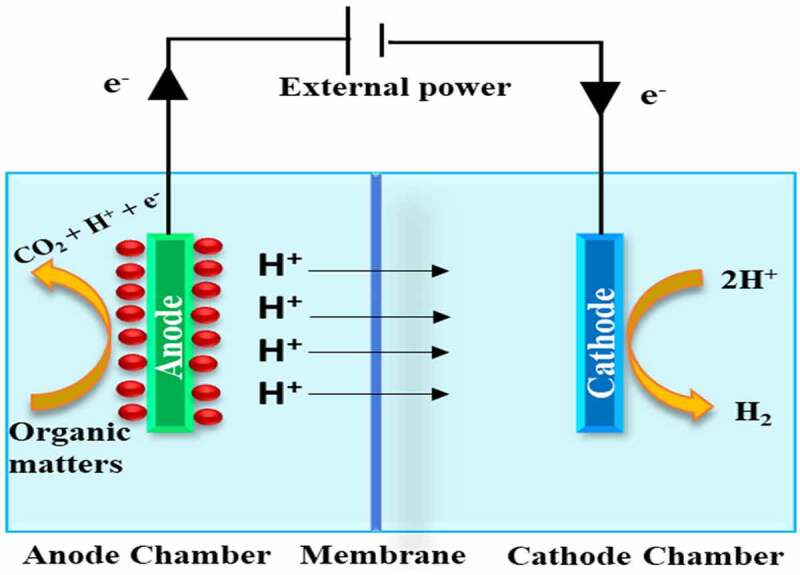
Diagrammatic representation of Double Chamber MEC.

The use of a double chamber in microbial electrolysis cells, with a membrane acting as a separator, may reduce organic material and bacterial crossover from the anodic chamber to the cathodic chamber, and it also contributes to the reduction of contaminants like CO_2_, CH_4_, and H_2_S [27]. Using a membrane also works as a separator to prevent any short-circuit. This is significant since a short length between the electrodes is needed to lower the internal strength. The double chamber draft was initially used as conceptual evidence of microbial electrolysis cells by two groups at approximately the same time frame. The transport rate of an H^+^ (proton) and/or OH^−^ (hydroxyl) all over the membrane is generally restricted in the neutral state, resulting in a pH fall all over the membrane [28]. The pH variation throughout the membrane may reduce the rate of H_2_ generation by inhibiting Electrochemically Active Bacteria (EAB) activity and increasing input strength. It was demonstrated that the double-chamber microbial electrolysis cell was not appropriate for recovering H_2_ from unbuffered wastewater as a result of the large pH drop. In double-chamber microbial electrolysis cells for less buffered effluent treatment, a new operation mode involving occasional polarity inversion was employed [29]. Figure 1 shows the diagrammatic representation of a double-chambered MEC.

**Figure 2.**
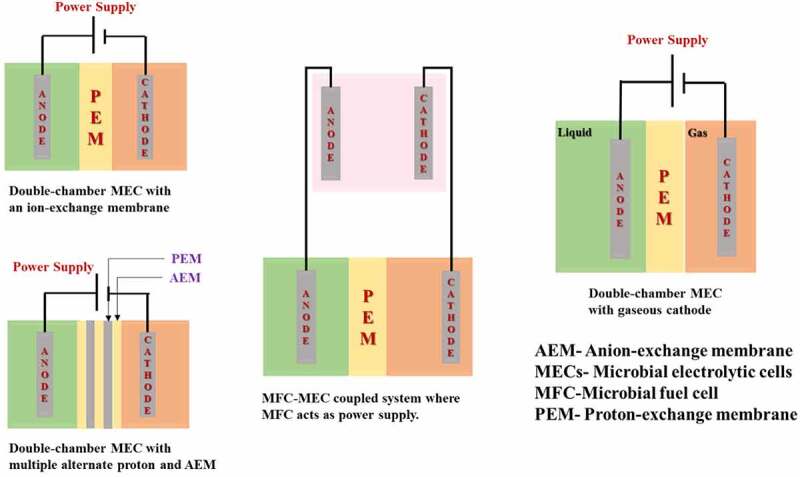
Schematics of different types of Double Chamber MEC.

Different forms of double-chamber microbial electrolysis cells have been created, namely cruse-type, barrels shaped, circular like a disk, and concentric tube-shaped, by reducing the distance between the electrodes and using a porous geotextile membrane as the separator, a flat-type microbial electrolysis cell was lately developed to reduce input resistance [30]. Figure 2 shows schematics of different types of Double Chamber MEC.

### 2.2 Single-chamber MECs system

**Figure 3.**
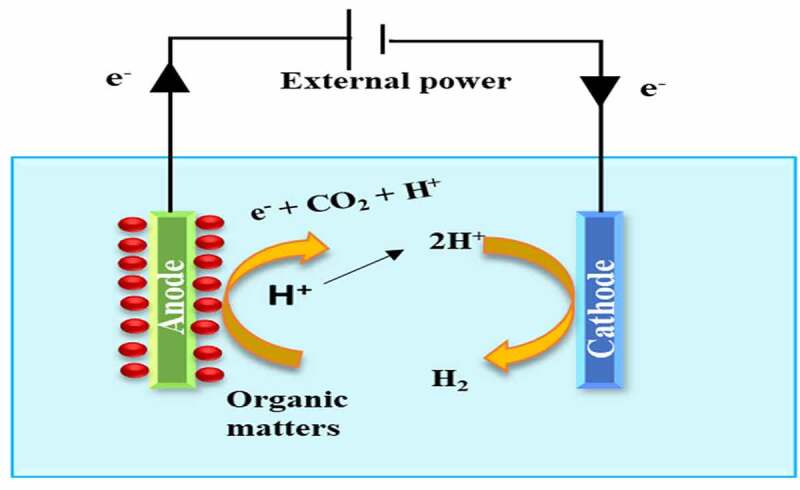
Diagrammatic Representation of Single Chamber MEC.

By eliminating membranes from two-chamber systems and dipping both the anode and the cathode into a similar solution of one chamber, a one-chamber microbial electrolysis cell was created. The usage of single-chamber microbial electrolysis cell can help to decrease membrane resistance-related losses [31]. It is simple to manufacture and autoclave, and it eliminates the problems associated with membranes, like fouling, deterioration, and massive price. The one-chamber microbial electrolysis cells have been created in a variety of designs, with the main structure constructed of diverse materials like a glass cruse and plastic block. Limited microbial electrolysis cells made from glass serum cruses can be used for the more-throughput bio-electrochemical investigation to choose the Electrochemically Active Bacteria (EAB) or possible cathode substances [28]. Figure 3 demonstrates the diagrammatic representation of Single Chamber MEC.

The use of produced H_2_ by methanogenic bacteria and the re-oxidation of H_2_ by Electrochemically Active Bacteria are the most difficult challenges in one-chamber microbial electrolysis cells. UV irradiation was used to impede methanogenesis [32]. Lately, the active gas harvesting approach was used to eliminate H_2_ consumption by using a vacuum and gas-permeable hydrophobic membrane to remove H_2_ quickly [29; 33]. There are numerous advantages to using gas-permeable membranes. By using bubble-less hydrogen diffusion, they may improve hydrogen transfer, increase hydrogen utilization efficiency, and reduce explosion hazards. Gas-permeable membranes can be combined with either suspended or connected growth systems. Through the membrane walls, hydrogen diffuses into the bulk liquid as it travels through the lumen [34].

## 3. Advantages and disadvantages of MEC technology

The MEC technology has proved to be an excellent process for resource recovery from the wastewater stream generated from various industries and posses several advantages over the traditional treatment methods. Some of the advantages of MEC Technology are like a) The MECs can be operated using a variety of substrates and the yields of H_2_ in most MECs are significantly higher, particularly in comparison to the fermentation process. Several acetates based MECs have produced up to 90% hydrogen yields, showcasing their potential in hydrogen production [35]. b) The MECs can be used in combination with the traditional anaerobic digesters to increase the production of CH_4_ in the anaerobic digester [36]. Insertion of MEC electrode into the anaerobic digester has proved to increase the production of CH_4_ from 60% to up to 98% and increased the carbon recovery to up to 55–56% [37]. c) The MECs can be integrated into the biorefinery setup to produce hydrogen and recover chemicals. Placement of the MECs just after the pre-treatment can help utilize substrates to produce hydrogen and other value-added products and increase the overall efficiency of the fermentation process. In a similar fashion, MECs can also be coupled with the fermentation process for a higher yield of hydrogen and products [38]. d) The potential of the MECs is also been evaluated for their role in the production of organic chemicals using the mechanism of microbial electrosynthesis. This can also be an alternate source for chemical generation [39]. Apart from the above-mentioned advantages, other advantages include reducing the use of fossil fuels which ultimately reduces greenhouse emissions, promotes the use of renewable substrates for energy generation, reducing the burden on the environment by processing organic wastes and upholding ecological recycling, etc [40].

However, there are some disadvantages of MEC Technology like a) Over a period of time, the yield of H_2_ decreases due to the several undesired electrons sinks in various metabolisms [41]. b) The setting up of a MEC reactor is determined by the configuration of the reactor, the materials used in its making, and the type of substrate that will be used in the reactor system. These configurations are set theoretically but the actual setting may vary from the theoretically set conditions and can alter the results [42]. c) For an efficient MEC, understanding the microbes and their relationship amongst themselves is essential to ensure the competition amongst the microbial species does not affect substrate utilization and product formation. Therefore, a thorough understanding of the microbes and their associated behavior is critical [43]. d) The use of MECs as a treatment for industrial waste can significantly reduce the organic constituents of the waste stream, however, it faces difficulty in meeting the standards set for the effluent discharge. Therefore, it is essential to refine this process to maximize its efficiency [44].

## 4. Coupling MEC with other technologies for value-added applications

### 4.1. MEC-anaerobic digestion coupled system

Anaerobic digestion occurs as a result of anaerobic microorganism metabolisms that degrade biodegradable materials and create biogas or CH_4_ gas [31]. This is a technique that has been utilized commercially for concurrent CH_4_ production and waste/effluent remediation [64]. Electrochemical techniques and anaerobic digestion can be merged for improved productivity, with stillage from a certain process utilized as feedstock in the earlier and energy retrieved from the earlier utilized in the latter [45]. In another study, an up-flow anaerobic sludge blanket reactor was compared to an up-flow anaerobic bio-electrochemical reactor for CH_4_ production from acidic distillery effluent. The up-flow anaerobic bio-electrochemical reactor was set to 300 mVolt, and both the up-flow anaerobic sludge blanket reactor and the up-flow anaerobic bio-electrochemical reactor were run in a constant state. The combination, i.e., up-flow anaerobic bio-electrochemical reactor, resulted in a considerably greater methane yield of 407 milliliters per gram CODr at 4.0-gram COD/L.day than up-flow anaerobic sludge blanket reactor (282 milliliters per gram CODr) [46]. By integrating a microbial electrolysis cell technology into the reactor, De Vrieze et al. attempted to assess the rational structure underlying the enhanced operation of anaerobic digestion. They found that adding electrodes and balancing them at 0.75 and 1.205 volt enhanced the stability of the anaerobic digestion treating treacle. Methane generation in the control reactors decreased to 50% of the starting rate (on day 91), whereas it stayed constant in the microbial electrolysis cell-anaerobic digestion reactors, implying a stabilizing impact. Surprisingly, when the electrodes from these reactors were placed in the control reactors, the CH_4_ output jumped three to fourfold. This demonstrated that the electrochemically active biofilm generated on the surface of the electrode, instead of the electric current, should have improved the constancy of anaerobic digestion. Integrating microbial electrolysis cells and anaerobic digestion is a great illustration of how microbial electrolysis cells may be utilized in a modular fashion to improve the productivity of current technology [47].

The underlying premise for organic substrate breakdown is identical in the anaerobic digestion and microbial electrolysis cell procedures. The ultimate end products differ because the ultimate electron acceptor is varied. In microbial electrolysis cells, CH_4_ can be created electrochemically as well as through fermentation processes. It has been proposed that combining these two procedures can help overcome the limits of separate methods. Cerrillo et al. observed that the microbial electrolysis cell system retrieved limiting products of the anaerobic digestion such as NH_3_, hence increasing the production of CH_4_ [48–50]. Since few traditional anaerobic digestion techniques have been marketed, the development of integrated anaerobic digestion – microbial electrolysis cell systems appears to be feasible in the coming years.

### 4.2. *MEC with anaerobic membrane bioreactor (MBR) (MBR and acidogenic*)

Membrane bioreactors are effluent remediation systems that combine a semipermeable membrane with a suspended growth bioreactor. The following are some examples of how membrane filtering can be included in bio-electrochemical systems: 1) Between the electrodes, as a separator, 2) In the anode/cathode section, there is an inner filtration element, or 3) Prior to or after the bio-electrochemical system, there is an outer remediation technique. More effective treatment has the benefits of integrated systems including increased energy efficiency, lower investment, less fouling, and/or long-term desalination [51]. Katuri et al. created the anaerobic electrochemical membrane bioreactor. A unique anaerobic remediation technology; a mix of microbial electrolysis cell and membrane filtration using nickel-based hollow-fiber membranes that are electrically conductive and porous [52]. The anaerobic electrochemical membrane bioreactor was used to treat low-organic-strength effluents and solutions, as well as to retrieve biogas. The nickel-based hollow-fiber membrane fulfilled 2 purposes: as a cathode electrode for producing hydrogen and as a membrane for filtering the purified water. At a 700-megavolt applied voltage, greater than 95% of the chemical oxygen demand (starting COD: 320 mg/L) was removed, and up to 71% of the substrate energy (methane-rich biogas, 83%) was retrieved. In addition, the anaerobic electrochemical membrane bioreactor had little membrane fouling than the control reactor, which was related to the generation of hydrogen bubbles, a lower cathode voltage, and a localized higher pH at the cathode surface. Although there are numerous advantages to integrating a membrane bioreactor with a microbial electrolysis cell, more information regarding energy production and consumption in the connected system is required.

#### 4.2.1 Acidogenic bioreactors

Acidogenic bioreactors can be paired with microbial electrolysis cells in the same way as anaerobic digestion and microbial electrolysis cells may be mixed. Babu et al. tested biohydrogen generation in a single chamber microbial electrolysis cell using CH_3_COO- (acetate), C_3_H_7_COO- (butyrate), and C_3_H_5_O_2_- (propionate) as substrates at varied voltages [53]. At 600 megavolts, the highest hydrogen production rate of 2.42 millimole/hour was reported, with around 53% of synthetic acids removed [54]. The same researcher’s group also paired an acidogenic bioreactor with microbial electrolysis cells to boost product retrieval and hydrogen production. microbial electrolysis cell was run at 3000 milligram/liter volatile fatty acid concentrations under various poised potentials, with the highest hydrogen production rate of 0.53 millimole/hour and 49.8% volatile fatty acid consumption observed at 600 megavolts. A unique bio-electro hydrolysis system based on self-inducing electrogenic activity was developed as a pre-treatment tool to boost hydrogen generation efficiency via the remediation of food waste in another research. Hydrolysis (1^st^ stage) was preceded by acidogenic fermentation for hydrogen generation (2^nd^ stage) in a two-stage coupled or hybrid system. Bio-electro hydrolysis produced more hydrogen (29.12 milliliter/hour) as a result of pre-treatment than the control (26.75 milliliter/hour). Furthermore, substrate breakdown was increased with the bio-electro hydrolysis-pre-treated substrate (a 52.42% reduction in chemical oxygen demand) as compared to the control (chemical oxygen demand removal of 43.68%) [55].

56,additionally developed a one-chambered microbial electrolysis cell with an acid-pre-treated biocatalyst for electro fermentation of wastewaters for further Hydrogen generation while simultaneously treating the wastewater. At 200 mVolt and 600 mVolt applied potentials, the effect of volatile fatty acid concentration (4000 milligrams/liter and 8000 milligrams/liter) on biohydrogen generation with simultaneous treatment was investigated. At 600 mVolt, the highest hydrogen production rate was 0.057 mVolt/hour, with a volatile fatty acid utilization of 68% [56]. As a result, by coupling microbial electrolysis cells with acidogenic reactors, wastewater from these reactors might be transformed into usable chemicals like hydrogen.

### 4.3. Thermoelectric micro converter-MEC coupled system

Industrial procedures, such as those in the automobiles and steel sectors, produce waste heat as a by-product, which thermoelectric converters can use as a source of energy, and this energy is known as thermoelectricity. Thermoelectricity changers work by using a temperature gradient in the medium, which can be liquid or other solid phases. Industrial processes produce a lot of heat, which can generate a temperature gradient that can help with thermoelectricity production. Cooler temperatures, on which there is little research, can also be used to capture thermoelectricity. It is more eco-friendly to retrieve waste heat from cold temperatures. The operation of microbial electrolysis cells for the creation of hydrogen necessitates only a little quantity of electrical energy. Thermoelectricity is a natural non-conventional resource [57]. As a result, combining microbial electrolysis cells with thermoelectricity can improve the long-term viability of the Hydrogen generation procedure. Chen et al. investigated the impacts of various temperature ranges on Hydrogen synthesis from CH_3_COO^−^ as a carbon source in a thermoelectric micro converter-microbial electrolysis cell linked system. The thermoelectric micro converters produced electric potential and were discovered to affect the systems’ hydrogen production rate. The voltage ranged between 170 mVolt and 830 mVolt depending on the temperature (between 35°C to 55°C). At 55°C on the hot side, the highest hydrogen generation of 0.16 m^3^ /day and output of 2.7 mol/mol CH_3_COO- were reported, with an average voltage of 700 mVolt and current density ranging from 0.28 to 1.10 ampere/meter square [29,57].

### 4.4 Dark fermentation and MFC–MEC coupled system

A dark fermentation reactor, 1 to 3 microbial fuel cells, and one microbial electrolysis cell made up the combined H_2_ generation system. Cellulose was continually fed to the fermentation reactor, and the wastewater was accumulated and used to feed the microbial fuel cells and microbial electrolysis cells. Using fermentation wastewater as a feed, two microbial fuel cells (each 25 milliliters) linked in series to a microbial electrolysis cell (72 milliliters) achieved a highest of 0.43 Volts, resulting in a hydrogen production rate from the microbial electrolysis cell of 0.48 m^3^ hydrogen/m^3^ /day and a yield of 33.2 millimole hydrogen/gram chemical oxygen demand eliminated in the microbial electrolysis cell [25; 29].

Microbial electrolysis cells and dark fermentation can be combined to increase total H_2_ production from a particular substrate [58,59,60]. Because the dark fermentation procedure has a thermodynamic limit (4 mol hydrogen/mol glucose), the remaining energy from the substrate can be utilized in microbial electrolysis cells to retrieve more H_2_. Acidogenic bacteria transform combined organic substrates to hydrogen and volatile fatty acids as a by-product of the dark fermentation procedures. These volatile fatty acids can also be used as electrogenic bacteria substrates in microbial electrolysis cells. This coupling boosts gross H_2_ yields while also allowing for high substrate transformation efficiencies. When combined organic wastes (like lignocellulosic wastes) are employed as substrates, the 2-stage coupling is advantageous. Even though such substrates can be utilized natively in microbial electrolysis cells, their generation rates and procedure efficiency are insufficient when compared to volatile fatty acids. The division of energy retrieve into two processes allows for more efficient regulation of each procedure. Nevertheless, in order to attain greater efficiency, the scale of microbial electrolysis cells used must correspond to the level of dark fermentation. Separating cell biomass and adjusting the potential of hydrogen during the transition from dark fermentation to microbial electrolysis cells is still a challenge for the combination of these systems. In addition, for constant H_2_ generation from the 2-stage procedure, appropriate structures are required [61,62].

Biological hydrogen generation from soluble organic components in effluents opens up the possibility of dark fermentation to make use of these previously unexplored resources. Furthermore, several thermodynamic hurdles make obtaining optimal outcomes via dark fermentation procedures difficult. Because of these thermodynamic constraints, many by-products such as CH_3_COO^−^ and C_4_H_7_O_2_ are generated instead of hydrogen, necessitating the addition of external energy to make the process thermodynamically possible. The highest of four moles of hydrogen and two moles of CH_3_COO^−^ are produced by dark fermentation of one mole of glucose. CH_3_COO^−^ can also be transformed to Hydrogen via a bio catalyzed electrolysis process. A possible output of 12 moles hydrogen/mole glucose might be achieved by combining the two methods.

### 4.5. *Microbial reverse-electrodialysis electrolysis cells (MRECs*)

Reverse electrodialysis uses a succession of cation exchange membrane and anion exchange membrane to produce electricity from the salinity gradient that occurs between ocean water and pure water. Each membrane produces voltage as a result of the chemical potential contrast between pure and salt water, and the overall potential of the system is the total potential contrast from all membranes [63,64]. Reverse electrodialysis systems typically employ a large number of stacked cells to achieve a high level of energy recovery. The procedure results in significant capital costs due to the vast number of membranes used, as well as increased energy disadvantages from pumping water via a wide variety of cell types. When microbial electrolysis cells are combined with the reverse electrodialysis system, a microbial reverse-electrodialysis electrolysis cell is formed, in which large overpotentials can be mitigated by the oxidation of organic material by anodic biocatalyst, while the voltage drop of microbial fuel cells can be enhanced because of the salinity-driven potential of the reverse electrodialysis stack. Microbial reverse-electrodialysis electrolysis cells were used by Cusick et al. to extract salinity-gradient energy from thermolytic NH_4_HCO_3_ solutions that produced minimum waste heat (greater than 40°C). Collecting salinity gradient energy from certain thermolytic solutions makes this procedure less reliant on the supply of saltwater and pure water. The restriction of the microbial reverse-electrodialysis electrolysis cell stack configuration with ammonium bicarbonate is N_2_ crossing from the stack into the anode chamber, which results in NH_3_ contamination of the anodic solution and thus saline water reduction. Future microbial reverse-electrodialysis electrolysis cells could benefit from bipolar membranes or a minimum-salt solution in the membrane stack closest to the anode to reduce the above-mentioned losses. The highest energy retrieval with CH_3_COO^−^ was found to be 30.5% with a power density of 5.6 Watt/m^2^ in research, which was 5 times higher than without the treatment stack. Furthermore, the reduction of power in the microbial reverse-electrodialysis electrolysis cell caused by the extra chamber near the anode might be mitigated by adding two additional membranes to boost the stack voltage. Zhu et al. developed a microbial reverse-electrodialysis electrolysis cell variant dubbed the microbial reverse-electrodialysis chemical-generation cell (MRCC). The energy generated by organic material (CH_3_COO^−^) and salt gradients were used by microbial reverse-electrodialysis chemical-generation cells to generate acid and alkali (saline water and river water imitative with various concentrations of NaCl). There was no need for an additional power supply because this system generated enough electricity (908 mWatt/m^2^) on its own. 1.35 millimole acid (pH 1.65 ± 0.04) and 0.59 millimole alkali (pH 11.98 ± 0.010) were produced during the fed-batch cycle performance [25,65].

66,created a one-of-a-kind approach for H_2_ generation by integrating a tiny reverse electrodialysis stack (5 membrane couples) with a microbial electrolysis cell, which they dubbed microbial reverse electrodialysis electrolysis cell [66]. Additional power supplies are not required in microbial reverse electrodialysis electrolysis cells since the energy for hydrogen generation comes from microbial oxidation of organic material in the anode and the saline differential between river water and seawater. During every fed-batch cycle, the microbial reverse electrodialysis electrolysis cell, which was built with 5 pairs of saltwater and river water cells, generated between 21 and 0.026 liters of gas. For an anode and cathode vessel, a cubic Lexan block with a barrels-shaped chamber (0.03 liter, 7 cm^2^ in cross-section) was utilized, with a glass pipe (0.02 L) affixed to the head of the cathode chamber to gather hydrogen. A microbial reverse electrodialysis electrolysis cell was formed by sandwiching 5 pairs of saltwater and river water cells between an anode-bearing exo-electrogenic bacteria and a cathode. At a saline ratio of 50, exoelectrogens provided an electrical potential from CH_3_COO^−^ oxidation and lowered the anode overpotential, while the backward electrodialysis stack contributed 0.5 to 0.6 Volt. For sea and river water flow rates scale from 0.1 to 0.8 milliliter per minute, the hydrogen production rate raised from 0.8 to 1.6 m^3^ – hydrogen/m^3^ -anolyte per day. The ratio of electrons utilized for hydrogen evolution to electrons liberated by substrate oxidation, known as hydrogen recovery, ranged from 72% to 86%. Energy efficiency ranged from 58% to 64%, based on changes in salt concentrations and the depletion of organic material [29]. Table 1 shows the integration of Microbial Electrolysis Cell with other technologies for energy production.

**Table 1.** Integration of Microbial Electrolysis Cell with other technologies for energy production

S. No	Integration Type	Type of Electrode	Applied Energy [Voltage/ Current)	Type	Hydrogen	Current or Power Density	Remarks	References
	1. Microbial Electrolysis Cell-Anaerobic Digestion coupled	Anode: Carbon felt Cathode: stainless-steel	0 mV	Double chamber	0.42 ± 0.05 m^3^ CH_4_/m^3^ /d	2.01 ± 0.63 A/m^2^	AD-MEC loop system was used	48
	2. Microbial Electrolysis Cell-Anaerobic Digestion coupled	Anode: Carbon brush Cathode: Ti/RuO_2_	0.0–0.8 V	Single chamber	CH_4_ at 0.4 V	Steady increase in current with applied voltage	To update CH_4_, AD-MEC was combined.	50
	3. Microbial Electrolysis Cell with Hydrogen Bioreactor [HBR]	Anode: Graphite plate Cathode: Graphite plate	0.6 V	Single chamber	0.53 mmol/h	N/A	At 0.6 V, HBR+MEC produced the best results.	93
	4. Thermoelectric micro converter-Microbial Electrolysis Cell coupled system	Anode: Plain CF Cathode: Carbon paper with Platinum.	0.17 to 0.83 V	Double chamber	0.16 m^3^ H_2_/m^3^ /d	0.28 to 1.10 A/m^2^	Thermoelectric micro-converter as power source	57
	5. Dark fermentation and Microbial Fuel Cell–Microbial Electrolysis Cell coupled system	Anode: Carbon brush Cathode: Platinum coated carbon cloth	0.33 to 0.47 V	Single chamber	0.48 m^3^ H_2_/m^3^ /d	52 A/m^3^ [MEC]	Combine MFC and forest organics in a stack.	58

## 5. Applications of the microbial electrolysis cell

### 5.1. Obtaining value-added products

#### 5.1.1. Hydrogen production

A viable treatment solution for improved H_2_ generation from food waste was determined to be a one-chamber microbial electrolysis cell with negative pressure control [67]. To obtain efficient H_2_ retrieve Food Waste was used as a substrate in a combined reactor that included one-chamber microbial electrolysis cell remediation and anaerobic digestion [68]. Throughout continuous anaerobic digestion-microbial electrolysis cell operation, some investigations used a combined reactor to integrate single-chamber H_2_ production (511.02-milliliter hydrogen gram 1 VS), which was greater than that attained by anaerobic digestion (49.39-milliliter Hydrogen gram 1 VS). In anaerobic digestion-microbial electrolysis cells, H_2_ recovery was 96% and electrical energy recovery was 238.7%, accordingly [69]. Key components of Food Waste [lipids, VFAs, carbohydrates, and protein] were studied to determine use of organic material to find the mechanism of H_2_ generation rise. The clearance efficiencies of proteins and carbohydrates in the dissolved phase in anaerobic digestion-microbial electrolysis cells were raised by four times and 2.3 times, significantly, as compared to anaerobic digestion treatment. Volatile fatty acids elimination by anaerobic digestion-microbial electrolysis cell was raised by 4.7 times, indicating that the anaerobic digestion reactor in combination with microbial electrolysis cell technologies increased the usage of the primary organic substances and hence improved H_2_ generation. As a result, study illustrates feasibility of minimizing Food Waste amounts while still producing bio-hydrogen. Microbial electrolysis cell technique is regarded as a resource-efficient solution for food Waste treatment with variety of engineering applications [69–72].

#### 5.1.2. Methane production

Anaerobic digestion in combination with a microbial electrolysis cell is anticipated to speed up CH_4_ synthesis from biomass hydrolyzate in microbial electrolysis cell-anaerobic digestion feeding with raw waste activated sludge and heat pre-treated waste activated sludge, accordingly, the methanogenesis efficiency and responsiveness of operational microbes [1]. The study described a low-cost adaptive technique for increasing CH_4_ productivity and output by using waste-activated sludge as a substrate for microbial electrolysis cell-anaerobic digestion. The CH_4_ production and productivity were increased by 9.5 and 7.8 times, respectively, when the applied voltage was 0.8 Volt greater than the standard open circuit (stage I), and by 6.3 and 6.2 times, respectively, when the voltage was returned to 0 Volt [73]. When raw waste-activated sludge was used in microbial electrolysis cell-anaerobic digestion, the hydrolysis-fermentation and acidogenesis were significantly boosted, leading to a greater synergy of acetogenic bacteria and hydrogenotrophic methanogens, as compared to the methods for enhancing methanogenesis with heat pre-treated waste activated sludge as substrate. As a result, microbial electrolysis cells could be employed as a viable option to boost operational microorganisms in order to produce high and steady CH_4_ generation in traditional anaerobic digestion while also lowering the greater operating costs associated with pre-treatment and voltage [16,74–76]

#### 5.1.3. Formic acid production

Formic acid (HCOOH) is a colorless, caustic acid that dissolves in water and a variety of other polar solvents. It is primarily utilized in the textiles, pharmaceuticals, and food industries, with the tanning and leather industries accounting for the majority of its usage in 2003 [77]. Meanwhile, its use as a supplement and protector in livestock feed has lately surpassed this, accounting for roughly 20% of worldwide HCOOH usage in 2019 [78]. Along with its ever-expanding spectrum of applications, the need for the chemical is expected to rise by about 6% in 2019. Because of its characteristics and ease of dehydrogenation, HCOOH can be utilized to store H_2_. HCOOH is typically produced through the oxidation of hydrocarbons (HCs), the hydrolysis of HCONH_2_, the synthesis of HCOO^−^ and the hydrolysis of HCOOCH_3_. CH_4_ and CH_3_OH can be used in the oxidation of hydrocarbons (HCs) to make HCOOH.

Hydrocarbon oxidation,

(1) $$CH_{3}OH\;+\;1\;/\;2\;O_{2}\to \;HCHO\;+\;H_{2}O$$

(2) $$HCHO\;+\;1\;/\;2\;O_{2}\to \;HCOOH$$

Formate-based production,

(3) $$2HCOONa\;+\;H_{2}SO_{4}\to \;Na_{2}SO_{4}+2HCOOH$$

(4) $${\left(HCOO\right)}_{2}Ca\;+\;H_{2}SO_{4}\to \;Ca_{2}SO_{4}+2HCOOH$$

Methyl formate hydrolysis,

(5) $$CH_{3}OH\;+\;CO\;\to \;HCOOCH_{3}$$

(6) $$HCOOCH_{3}+H_{2}O\;\to \;CH_{3}OH\;+\;HCOOH$$

**Figure 4.**
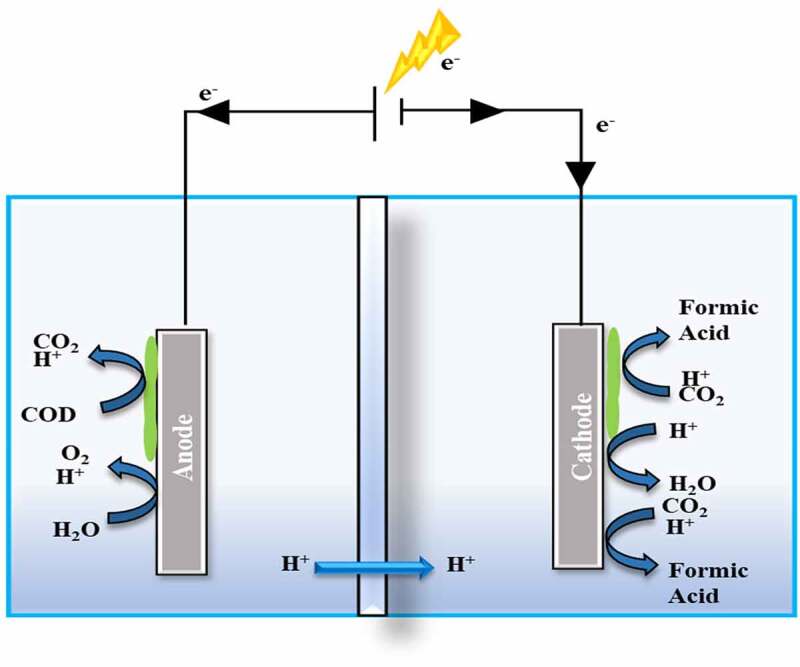
Formic acid generation using biotic and abiotic electrocatalysis.

In a two-step procedure, CH_3_OH is oxidized to CH_2_O, which is then oxidized to HCOOH [see Eq. (1) and (2)]. However, employing heterogeneous catalysts, CH_4_ is oxidized to yield HCOOH. Although outputs are modest, employing CH_4_ is favorable since oxidation happens at cooler temperatures (at 60°C). until the end of the 19th century, CH_2_O hydrolysis was a common method of producing HCOOH throughout Europe. The use of NH_3_ and H_2_SO_4_, as well as the production of (NH_4_)_2_ SO_4_, rendered the technique less cost-effective. HCOONa [see Eq. (3) in the following section] and Ca (HCO_2_)_2_ [see Eq. (4) in the following section] are commonly used in formate generation, with H_2_SO_4_ or H_3_PO_4_ used for acidolysis. When comparing the various methods HCOOH can be made traditionally, hydrolysis of HCOOCH_3_ is presently the most used method. Around 90% of all HCOOH installation manufacturing plants are located along this path. It is produced in two-stage procedures in which 95% CO and 30% CH_3_OH are combined to make HCOOCH_3_, which is subsequently hydrolyzed to give HCOOCH [see Eq. (5) and (6)] [78–80]. Figure 4 demonstrates the formic acid generation using biotic and abiotic electrocatalysis

#### 5.1.4. Hydrogen peroxide production

Microbial electrolysis cells can also create hydrogen peroxide (H_2_O_2_), an essential industrial chemical. The viability of producing hydrogen peroxide by combining microbial oxidation of organic materials in the anode with oxygen reduction in the cathode of microbial electrolysis cells has recently been established. This system was able of creating hydrogen peroxide at a rate of 1.17 millimole/L/hour in the aerated cathode with an applied voltage of 0.5 Volt, leading to a massive efficiency of 83% based on CH_3_COO^−^ oxidation. Hydrogen peroxide synthesis in microbial electrolysis cells uses far less energy than in typical electrochemical methods, with 0.93 kWh/kg hydrogen peroxide reported in the research [81]. More work should be placed toward improving hydrogen peroxide concentration for this innovation to mature. The highest hydrogen peroxide concentration currently achievable in microbial electrolysis cells is 0.13 wt.%, which seems to be an order of magnitude below the expected level for actual industrial ramifications [47,82,83]. Table 2 shows value-added products obtained by employing microbial electrolysis system.

**Table 2.** Value-added products obtained by employing microbial electrolysis system

S.No.	Value Added Products	Substrate	Microbial Electrolysis Cell reactor	Voltage Applied	References
1.	Hydrogen	Raw food waste	Microbial Electrolysis Cell-Anaerobic Digestion reactor Single chamber	0.8 V	(69]
2.	Methane	Raw waste Activated sludge	Microbial Electrolysis Cell-Anaerobic Digestion reactor Single chamber	0 V, 0.6 V, 0.8 V	[16]
3.	Formic Acid (HCOOH)	-	Microbial electrolysis desalination and chemical-production cell	1.2 V to 2.4 V	[77]
4.	Hydrogen peroxide (H_2_O_2_)	Organic matter	Two-chamber Microbial Electrolysis Cell	0.5 V	[34]

### 5.2. Removal of complex pollutants with simultaneous resource recovery

#### 5.2.1. Organic pollutants

Most organic pollutants, like nitrobenzene, can be eliminated in microbial fuel cells, but in microbial electrolysis cells with a little quantity of energy supply, the elimination can be greatly boosted [X. 70, 84–89]. To decrease nitrobenzene, without membrane, an up-flow microbial electrolysis cell-type reactor was created. With an outer voltage source of 0.5 Volt, up to 98% of nitrobenzene was eliminated in the cathode region, leading to the highest extraction rate of 3.5 mol/m^3^ /day. C_6_H_5_NH_2_ was the primary product of nitrobenzene destruction, with a generation rate of 3.06 mol/m^3^ /day. This procedure required under 0.075 kWh per mol nitrobenzene in terms of total energy [90].

Chlorophenols (CPs) are hazardous, bio-refractory, and tough to break down in the native environment type of chlorinated contaminants. The possibility of ClC_6_H_4_OH (4-chlorophenol) removal in double-chamber microbial fuel cells and microbial electrolysis cells was examined by Wen et al. With only a little quantity of electricity produced, the 4-chlorophenol (ClC_6_H_4_OH) reduction procedure was possible in the double-chamber microbial fuel cell. The dichlorination accuracy of 4-chlorophenol at the cathode, on the other hand, was just 50.3%. When the reactor was performed in microbial electrolysis cell mode with 0.7 Volt voltage input, it increased to 92.5%. With an energy expenditure of 0.549-kilowatt h/mol 4-chlorophenol, the highest dichlorination rate achieved 0.38 mol per m^3^ /day. The energy consumption of microbial electrolysis cells for dichlorination was significantly lesser than that of standard electrochemical techniques, which needed roughly 1.17-kilowatt h/mol 4-chlorophenol. BTHs (benzothiazole derivatives) are a class of xenobiotic heterocyclic compounds that are poisonous and tough to decompose in nature. In microbial electrolysis cells, Liu et al. looked at the viability of removing Benzothiazole derivatives. In a sulfate-reducing bacteria (SRB)-enhanced microbial electrolysis cell system, Miran et al. examined the chlorinated phenol contaminant (ClC_6_H_4_OH (4-CP)) remediation. The results demonstrated that the sulfur-reducing bacteria-enriched microbial electrolysis cell effectively eliminated 4-chlorophenol and allowed for further dichlorination of dechlorinated compounds [88,91,92].

#### 5.2.2. Inorganic pollutants

Organic decomposition in the microbial electrolysis cell has been observed to be significantly improved by bio electrochemical augmentation of sulfate reduction [93]. In the anode chamber, organic substrates were oxidized, while in the biocathode, sulfate served as an electron acceptor. According to Li et al., microbial electrolysis cells can increase anaerobic acidogenesis by processing sulfate-containing effluent, and microbial electrolysis cell-based acidogenesis can achieve better sulfate removal even at greater sulfate loadings [94,95]. Furthermore, Dong et al. employed a one-chamber microbial electrolysis cell with 600 milligram L-1 potassium sulfide concentrations in which the sulfide removal (K_2_S) rate achieved 80.7% and the main bacteria discovered was *Geobacter* (up to 7.35%) [96]. Wang et al. introduced electron transfer efficiency via neutral red that worked as the electron transfer facilitator to increase the electrocatalytic activity of microbial electrolysis cells on sulfate elimination, and the sulfate reduction in this reactor achieved 79% [97]. Exploring the ideal working conditions in real effluents is one of the difficulties of microbial electrolysis cells for sulfate elimination.

Sulfate (SO_4_^2-^), perchlorate (ClO_4_^−^), and nitrate (NO_3_^−^) are among the inorganic contaminants that can be eliminated at the cathode of microbial electrolysis cells [98,99]. Sulfate reduction as an electron acceptor has previously been observed in a microbial electrolysis cell using a bacteria-catalyzed cathode [100]. Sulfate reduction was not seen in the absence of voltage, but it improved when the voltage was introduced, reaching over 50 gm SO_4_^2-^/m^3^ /day at 1.4 Volt. A minimum voltage of 0.7 Volt is required for such a procedure [101]. Because of the increase in the potential of hydrogen in the cathode, the final product of sulfate decrease was sulfide, which was retained in the ionic form. Organic material oxidation and sulfate (SO_4_^2-^ reduction procedures are physically isolated in this method, preventing methanogenic bacteria from competing for electrons [102]. Because no additional organic material is required, this approach can be used to remediate sulfate-polluted groundwater without affecting the biostability of potable water. The significant pH differential between the anode and cathode, as well as the less conductivity of groundwater, are the method’s principal drawbacks. In this case, treating industrial effluents with greater conductivity could result in improved remedial efficiency [101,103]. Due to the hazardous effects and challenging breakdown features of heavy metals, remediation of heavy metals polluted effluent has received a lot of attention lately. Microbial electrolysis cells are a cost-effective and reliable technology for treating effluent-containing heavy metals as nickel ions. Microbial electrolysis cells had a three-fold better Nickel (II) ion extraction efficiency than traditional electrolysis cells and microbial fuel cells. The applied voltage and the starting nickel concentrations in the microbial electrolysis cells are 2 important parameters in Nickel ion elimination. According to X-ray diffraction quantifications, metal nickel was detected as an outcome of nickel ion reduction on the cathode electrode. For the remediation of nickel ion-containing effluents, microbial electrolysis cells could be a viable alternative to traditional electrolysis. Numerous problems must be overcome in order to make this technique widely accessible. The system’s lengthy durability must be investigated, as the production of metal nickel on the surface of the cathode may impact the cathode reaction activity, which in turn may affect system efficiency [104].

## 6. Life cycle assessment of microbial electrolysis cells

Life cycle assessment (LCA) is an instrument to assess the potential impacts on the environment and human health that a product can have. This method follows a cradle to grave approach and assesses not only the impacts of the product but also the impact of the acquisition of raw material, manufacturing process of the product, the transportation of the product, and the disposal of the waste generated during the manufacturing and ultimately the disposal of the product at the end [105]. The LCA assesses the global warming potential, eutrophication potential, ecotoxicity, acidification potential, and the potential to harm the human health, of the manufacturing process. The LCA can help identify the stages of the process that are more potent for causing environmental damage and efforts can be made to reduce them. The LCA is an important tool for manufacturers and decision-makers to make environmentally sound decisions concerning the manufacturing processes [106].

Since the use of MECs for the treatment of wastewater is a relatively newer technology, LCA is an important tool to prevent any unplanned outcomes that may rise from the new technologies. The LCA of MECs should include the evaluation of energy, financial flow, and their potential to emit greenhouse gas emissions. The main end product of the MEC is mostly hydrogen. The storage and subsequent transport of the hydrogen must also factor in as an important parameter for the LCA. This is important because it is extremely difficult to store or transport hydrogen at atmospheric pressure and hence a large amount of energy is required to compress it along with a storage facility specific to the storage of hydrogen [107].

## 7. Role of microbial electrolysis cells toward circular bioeconomy

The world is encountering a new wave of urbanization, which has had serious consequences on natural resources and the environment [108–111]. Concerns about rising energy demand and the generation of massive amounts of waste are pervasive [112,113]. For example, sewage produced at a rate of 40 billion liters per day results in more than 10 million tonnes of extra sludge and more than 20 million tonnes of CO_2_ emissions when incinerated. In this regard, humanity has only scratched the surface of the potential of biogenic waste [114]. These old ways of dealing with waste not only lack system integration but also cause negative impacts on the environment on a regional and global scale [115].

The circular economy concept aims to keep the value of goods, materials, and resources in the economy for as long as humanly possible while reducing waste generation [116–119]. According to the ‘Ellen MacArthur Foundation’, the circular economy is a restorative, regenerative paradigm in which ‘nothing is lost and everything feeds a new cycle.’ The cascading use of biomass (Biorefinery) substantially overlaps with the circular economy concept and is largely a part of it [120]. The primary goal of the bioeconomy and circular economy is to achieve better resource efficiency, resulting in a more sustainable and resource-efficient planet that relies primarily on recyclable items, hence lowering the carbon footprint [121–123].

Although much work and study have been done in recent years to develop green technologies that would aid in the achievement of a circular economy, there is still much work to be done [124,125]. As a result, greater investment in research and development is needed to develop more efficient circular technologies [126, 127, Renuka et al., 202; 128].

## 8. Technical challenges and prospects of microbial electrolysis cells

Microbial electrolysis cells (MECs) have the promise to be a long-term sustainable wastewater treatment technology. However, it encounters bottlenecks during scale-up. As a result, fresh scale-up experiences are needed to learn about crucial difficulties in MEC designs. The following are some of the primary obstacles that MECs encounter in scaling up:

### 8.1 Industrial feasibility

Despite MECs’ wide range of applications and promising future, industrial feasibility remains a major barrier, and system upscaling is necessary to evaluate this technology [66]. Recently, the scaling-up of MECs from bench to pilot-scale for production of hydrogen was described. Even though the electric energy recovery was greater than 70%, the Coulomb efficiency and the rate of hydrogen were much lower than the maximum value reported in lab-scale research, emphasizing the need for future tuning. Additionally, several MEC applications, like pollutant removal, chemical synthesis, and recovery of metal, have yet to be scaled up from a lab scale [129,130].

### 8.2 Economic challenges

Scaling up raises two important economic challenges: the materials of the electrode and reactor design. Efforts should be aimed toward the development of low-cost reactors capable of competing with a variety of different energy-generating and wastewater-treatment technologies. This, in turn, will have the additional benefit of supporting the phenomena of cost reduction with increasing manufacturing units, thus enhancing the practicability of this technology and favoring the development of more efficient MEC reactors. Cost-effective anode materials capable of efficient electron transfer from bacteria to the electrode in Microbial Fuel Cells, such as carbon fibre brush or activated carbon, might be a suitable alternative for anodes of MECs. Similarly, as a cathode catalyst, biocathode may be a promising alternative to noble metals. This is due to its low cost, excellent stability, and ecologically sound qualities. However, the effectiveness of the biocathode in pilot-scale activity remains unknown [43,131].

### 8.3 Process control and monitoring

Process control and monitoring is another significant area that has received little attention but it does have the ability to propel this technique forward on its path to practical use [132]. To attain this goal, a collaborative effort of engineers, microbiologists, electrochemists, and others would be required to understand the interactions between the many parameters and factors that may influence MEC function [133].

## 9. Conclusions

Microbial Electrolysis Cells show great potential in not only reducing the pollution quotient in the wastewater stream of the industry but also helping in aiding in the resource recovery from the industrial wastewater. This system not only helps in reducing biodegradable matter in the stream but also helps in generating electricity and hydrogen which is used as a clean fuel due to its nonpolluting properties. Due to its increased demand globally, the use of MEC has proved to be an efficient source of hydrogen generation. This system is energy positive and carbon negative and its integration with traditional technologies leads to higher output and refined processes. However, there are certain shortcomings in this process as well like the competition amongst the microbes, structural shortcomings, etc. and an extensive study and research needs to be directed in this regard to increase the efficiency of the process and maximize the yield produced in the process. Efforts to enhance reactor design and substrate selection can be done to maximize the efficiency of the setup. The MEC technology can prove to be a great asset for energy generation and resource recovery and can significantly help in reducing the dependence on conventional sources of energy like fossil fuels which are also polluting in nature. This technology can therefore pave a new way for energy generation and help meet the global energy requirement.
